# State-of-the-Art and Opportunities for Forward Osmosis in Sewage Concentration and Wastewater Treatment

**DOI:** 10.3390/membranes11050305

**Published:** 2021-04-21

**Authors:** Xing Wu, Cher Hon Lau, Biplob Kumar Pramanik, Jianhua Zhang, Zongli Xie

**Affiliations:** 1 CSIRO Manufacturing, Clayton South, VIC 3169, Australia; Xing.Wu@csiro.au; 2 School of Engineering, The University of Edinburgh, Edinburgh EH9 3FB, UK; Cherhon.Lau@ed.ac.uk; 3 School of Engineering, RMIT University, Melbourne, VIC 3000, Australia; biplob.pramanik@rmit.edu.au; 4 Institute for Sustainable Industries and Liveable Cities, Victoria University, Melbourne, VIC 8001, Australia; Jianhua.Zhang@vu.edu.au

**Keywords:** forward osmosis, sewage concentration, wastewater treatment, water recovery

## Abstract

The application of membrane technologies for wastewater treatment to recover water and nutrients from different types of wastewater can be an effective strategy to mitigate the water shortage and provide resource recovery for sustainable development of industrialisation and urbanisation. Forward osmosis (FO), driven by the osmotic pressure difference between solutions divided by a semi-permeable membrane, has been recognised as a potential energy-efficient filtration process with a low tendency for fouling and a strong ability to filtrate highly polluted wastewater. The application of FO for wastewater treatment has received significant attention in research and attracted technological effort in recent years. In this review, we review the state-of-the-art application of FO technology for sewage concentration and wastewater treatment both as an independent treatment process and in combination with other treatment processes. We also provide an outlook of the future prospects and recommendations for the improvement of membrane performance, fouling control and system optimisation from the perspectives of membrane materials, operating condition optimisation, draw solution selection, and multiple technologies combination.

## 1. Introduction

Freshwater is one of the scarcest resources in the world, and water shortage has become a serious problem with the growth of population and urbanisation [1,2]. In order to mitigate the negative impact of water shortage, water mining from wastewater was proposed by Lutchmiah et al. [3]. In this concept, wastewater is a resource for water, nutrients, and energy [3,4]. Various technologies such as advanced oxidation, activated carbon adsorption [5], anaerobic digestion (AD), and coagulation/flocculation (CF) have been applied to recover freshwater and nutrients from different types of wastewater, including sewage, landfill leachate, and textile wastewater [6]. However, one of the major challenges of nutrients and energy recovery from wastewater is the low-strength nature of wastewater, which not only impacts the recovery efficiency but also increases the construction cost for building reactors with large volumes [4]. Therefore, it is critical to find an effective approach to increase the concentration of matters such as chemical oxygen demand (COD) and nutrients (nitrogen and phosphorus) [4,7]. Membrane-based technology including microfiltration (MF), ultrafiltration (UF), nanofiltration (NF), and reverse osmosis (RO) have been studied and applied for various sources of wastewater treatment, including landfilling leachate [8,9] and textile wastewater [10,11]. However, the UF membrane has a low rejection toward small-size particles [12]. NF and RO processes still face challenges, such as high energy requirements, low water recovery rates, and serious membrane fouling [13,14]. For example, a study reported an average energy requirement of 1.2–1.5 kW/m^3^ to treat the pre-treated wastewater by a RO system [15].

**Figure 1:**
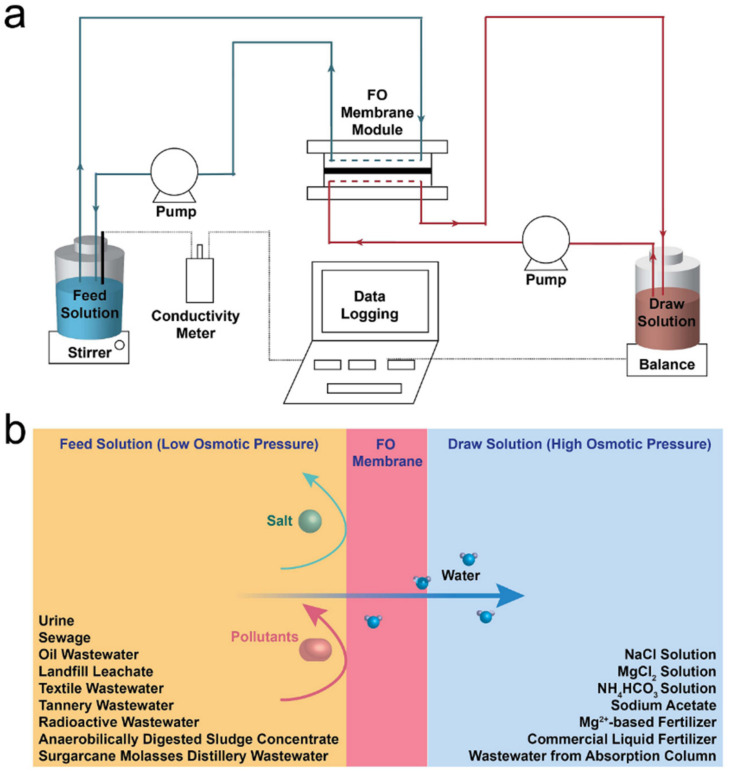
(**a**) Schematic of FO performance evaluation rig set-up, adapted from [33], and (**b**) major wastewater sources and draw solutions reported in FO-based wastewater treatment.

Recently, forward osmosis (FO) membrane-based technology has shown a growing interest in desalination [16,17], power generation [18], food concertation [19], wastewater treatment, and resource recovery [20,21]. During the FO process, water molecules spontaneously transfer through a semipermeable membrane driven by an osmotic pressure gradient between feed solution and draw solution (Figure 1a). Due to no requirements of hydraulic pressure, the FO process shows advantages such as low energy cost (0.02 kWh/m^3^ under certain operational conditions) [7], low fouling tendency, loose fouling layer, and reduced cleaning frequency [22]. Moreover, a FO membrane has a similar pore size to a RO membrane and good rejection of salts, heavy metals [23], and dyes [24]. Therefore, the FO process can be directly used to treat raw wastewater without complex pre-treatment processes [25,26].

So far, many cases about the application of the FO process as a single treatment process and hybrid systems integrating FO and other technologies have been investigated for treating various types of wastewater, such as sewage, landfilling leachate, and radioactive wastewater (Figure 1b). Besides the conventional inorganic draw solutes such as NaCl and MgCl_2_, several new types of draw solutions such as liquid fertilisers [27,28] and wastewater from absorption column [29] have been reported. However, challenges including membrane fouling, internal/external concentration polarisation, salt accumulation in the feed solution, and system operational condition optimisation still exist in FO technology. The FO process is a complex process, where the concentration of feed solution, dilution of draw solution, and reverse diffusion of solutes occur at the same time. Moreover, the performance of the FO system is associated with many factors, including FO membrane properties, areas of application, and operation conditions. Although there are a number of comprehensive reviews reported in recent years, they mainly focus on the fabrication and modification methods of FO membranes [30], the utilisation of FO membranes for desalination [31], and the concentration process [32]. In this work, we aim to provide a concise review of the latest development of applying FO technology for sewage concentration and wastewater treatment and focus on the interaction among membrane materials, feed solution properties, draw solution properties, and system operating conditions during the application. In addition, current challenges and recommendations for the improvement of FO systems, fouling control, and system design are discussed.

## 2. Deployment of FO as a Single Treatment Process

Due to the low membrane fouling tendency and high selectivity properties of FO membranes, the FO has been applied as a single treatment process to treat different types of wastewater. Some typical application cases are shown in Table 1. The major aims of the FO process include dewatering or concentrating feed solutions and diluting draw solutions, rather than producing water directly from wastewater. The performance of the FO system is related to many factors, including membrane properties (i.e., permeability, selectivity, and anti-fouling property), properties of draw solutions, and operating conditions of the FO system. In this section, the application of the FO as a single treatment process for sewage concentration and wastewater treatment is discussed. In addition, current research about the performance optimisation of the FO system is reviewed.

Sewage concentration is one of the mainstreams of the FO application in wastewater treatment. After the FO process, the sewage is featured with concentrated organics and is easier for further treatment [3,4]. Lutchmiah et al. [3] evaluated the performance of FO membranes in municipal wastewater concentration by using NaCl and MgCl_2_·6H_2_O as draw solutes, respectively. Although the water flux of the FO membrane was reduced when treating sewage instead of DI water, it was stable during the 6 h continuous testing. It was also found that the fouling layer on the membrane surface was thin and loose, which could be cleaned by simple water rinsing. Their result confirmed the low fouling tendency of FO membranes and indicated that the fouling was reversible in the FO process. Yang et al. [34] evaluated the municipal concentration performance of FO membranes by using synthetic seawater concentrate as the draw solution. After 24 h of filtration, the FO membrane showed good concentration efficiency for COD, ammonium nitrogen (NH_4_^+^-N), total nitrogen (TN), and total phosphorus (TP) (Table 2).

The stability of sewage concentration by FO membranes was further investigated for a long term by Wang et al. [4] in a pilot-scale FO system. During the 51-day experiment, a spiral-wound cellulose triacetate (CTA) FO membrane showed good rejection of chemical oxygen demand (COD, 99.8% ± 0.6%) and TP (99.7% ± 0.5%). However, the FO membrane had relatively low rejection of ammonium (48.1% ± 10.5%) and TN (67.8% ± 7.3%). This was likely due to the bidirectional diffusion of ammonium in the feed solution and sodium cations in the draw solution through the membrane [39].

Besides the sewage treatment, the feasibility of the FO process for a variety of wastewater types is also investigated. These wastewater sources include anaerobically digested sludge filtrate post-thermal hydrolysis process (THP-AD) [35], sugarcane molasses distillery wastewater containing complex phenolic compounds (melanoidins and polyphenols) [36], and radioactive wastewater containing harmful radionuclides (Co, Sr, and Cs) [23]. In these works, the FO membrane exhibited good rejections of melanoidins (90%), antioxidant activity (96%), and COD (84%) [36], low nuclide fluxes for Co (1.54 mg/m^2^h), Sr (10.22 mg/m^2^h), and Cs (15.63 mg/m^2^h) [23], and good dewatering performance (5 times concentration in volume) [35].

### 2.1. Selection of Draw Solutes

Sodium chloride (NaCl) has been widely used as a draw solute in the FO process due to its high osmotic pressure and ease to recover [40]. However, when FO is used for sewage concentration as the pre-treatment step of some biological processes, such as the activated sludge process, the salinity accumulation in the feed solution caused by the reverse NaCl diffusion reduces the water flux of FO membranes and hence affects the quality of sludge or retention feed solutions. Therefore, the selection of a suitable draw solute with a high osmotic pressure and a low back-diffusion rate is critical [41]. During the FO process, the feed solution will be concentrated, and the draw solution will be gradually diluted. Utilising the dilution process, Xie et al. [27] employed a commercial liquid fertiliser (containing K^+^, NH_4_^+^, and PO_4_^3−^) as the draw solution to recover clean water from raw sewage. The advantage of this process is that the liquid fertiliser diluted by the recovered water can be directly used for irrigation. By optimising the temperature, cross-flow rate, and liquid fertiliser concentration, the FO system achieved a high water recovery rate (>80%) and a stable diluted liquid fertiliser. The feasibility of using NH_4_HCO_3_ as a draw solution solute was investigated by a study recovering water from oil sand process-affected water (OSPW) [37]. By using 4 mol/L NH_4_HCO_3_ as a draw solution, the FO system achieved 85% water recovery, and high rejection rates (>70%) of F^−^, NO_2_^−^, Br^−^ ions, and Al, Ca, Fe, Sr, Mo, and Ba metals. Moreover, by applying backwash, the CTA-FO membrane achieved 77% water recovery. In a study reported by Soler-Cabezas et al. [38], the FO process was applied to concentrate the nitrogen in anaerobically digested sludge concentrate (ADSC). Results indicated that the industrial effluent (i.e., residual stream from an absorption process for ammonia elimination) could be applied as an effective draw solution. By using cellulose triacetate on radio-frequency-weldable nonwoven support (CTA-NW) FO membranes and using the industrial effluent as the draw solution, the NH_4_^+^-N concentration in ADSC increased from 824 to 1172 mg/L after the 70-h experiment. The advantage of using the industrial effluent as the draw solution was that it was not necessary to regenerate the diluted draw solutions [42]. Besides, in a study by Ansari et al. [41], the sewage concentration performance of FO membranes was compared by using NaCl, sodium acetate, and disodium ethylenediaminetetraacetate dihydrate (EDTA-2Na) as draw solutes. The result indicated that both sodium acetate and EDTA-2Na solutes effectively mitigated excessive salinity build-up in the pre-concentrated wastewater due to their lower reverse solute fluxes.

### 2.2. Effect of Process Parameters and Fouling Control

The performance of the FO filtration system is also related to process parameters such as temperatures of feed solution and draw solution. Additionally, fouling is one of the major barriers for all membrane processes. Therefore, fouling control is vital for improving the efficiency of FO processes, which could be achieved by tuning cross-flow velocity, optimising draw solution concentration, applying membrane cleaning processes, and fabricating FO membranes with good antifouling properties. During the design of the FO filtration process, it is necessary to consider all these factors comprehensively since there are some trade-offs and synergistic effects among them.

The temperatures of feed solution and draw solution can directly influence the thermodynamic properties of solutions [27,34], and consequently impact the water flux of FO membranes [43]. To be specific, the high feed and draw solution temperatures can reduce the solution viscosity and increase the water diffusion rate across membranes, resulting in higher water flux [44,45,46]. In a study about municipal wastewater treatment [34], the water flux of FO membrane decreased from 18.2 to 10.9 L/m^2^h when the temperatures of feed solution and draw solution reduced from 35 to 15 °C. In another study by Xie et al. [27], they observed that the membrane water flux increased four-fold as the temperatures of feed solution and draw solution increased from 5 to 45 °C. However, with the increase of solution temperature, the reverse solute diffusion also increased, which indicated that the trade-off between permeability and selectivity should be taken into account [34,47].

**Figure 2:**
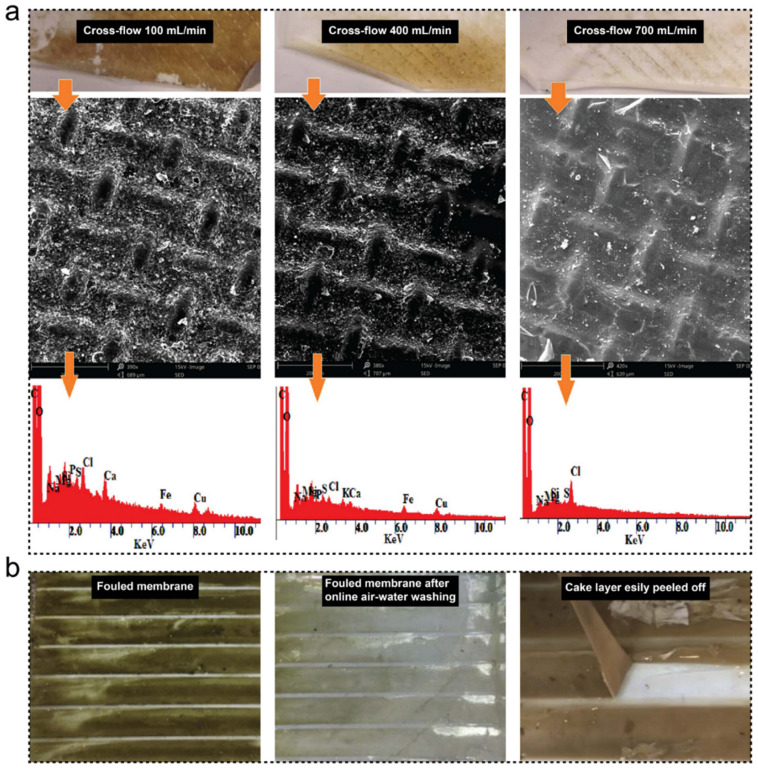
(**a**) Fouling behaviours of CTA-FO membranes with different cross-flow velocities, reprinted with permission from [48], and (**b**) photographs of the fouled membrane (**left**), fouled membrane after online air-water washing (**middle**), and visualisation of the easily peeled-off cake layer, reprinted with permission from [35].

The cross-flow velocity is another important factor in the FO system operation. Yang et al. [34] observed an increased water flux of the FO membranes from 14.9 to 18.2 L/m^2^h and a smaller water flux reduction rate by increasing the cross-flow velocity from 140 to 280 mL/min. The major reason for this improvement was that a high cross-flow velocity could provide positive effects on the mitigation of external concentration polarisation (ECP) and the control of membrane fouling during wastewater treatment processes, which consequently improved the water flux of FO membranes [34,41]. Lotfi et al. [48] investigated the fouling behaviour of FO membranes under different cross-flow velocities. The result indicated that a fouling layer on the membrane is more severe at low cross-flow velocity (100 mL/min). Nonetheless, no significant fouling on the membrane surface was observed after a long-term (three days) fouling test at the cross-flow velocity of 700 mL/min (Figure 2a). The impact of cross-flow velocity on fouling control and water flux improvement are based on two reasons. Firstly, the aggravation of foulants on FO membrane surface can be reduced by higher shear stress provided by a high cross-flow velocity. Secondly, the ECP can be mitigated by the enhancement of back-diffusion offered by a high cross-flow velocity, which not only improves the water flux but also increases the membrane cleaning efficiency [49,50,51].

In the study reported by Gao et al. [25] about municipal sewage concentration by CTA-FO membranes, they found that the membrane fouling behaviour and the water flux were affected by the concentration of draw solution. The result indicated that a high draw solution concentration could aggravate membrane fouling. However, due to the loose adhesion of foulants on the membrane surface, most of the foulants on the FO membrane can be washed off by a physical cleaning process (i.e., 15 min online air-water washing) and the water flux of membrane could be recovered up to 90% of a new membrane. Additionally, the water flux recovery rate can be further improved to more than 96% while an in situ chemical cleaning process (i.e., 30 min washing with 1% NaClO solution) was conducted after the physical cleaning. Similar results were reported in the study by Yang et al. [34], in which the flux of the FO membrane was recovered up to 94% post-treatment of raw sewage after applying 1 h physical membrane cleaning process. Chang et al. [35] compared the efficiency of three different membrane cleaning processes (online water washing, online air-water washing, and offline washing) in FO processes. The results showed that the online air-water washing exhibited an effective surface cleaning performance (Figure 2b), and the cake layer on the FO membrane surface could be easily peeled off, indicating the good fouling resistance of the FO process.

**Figure 3:**
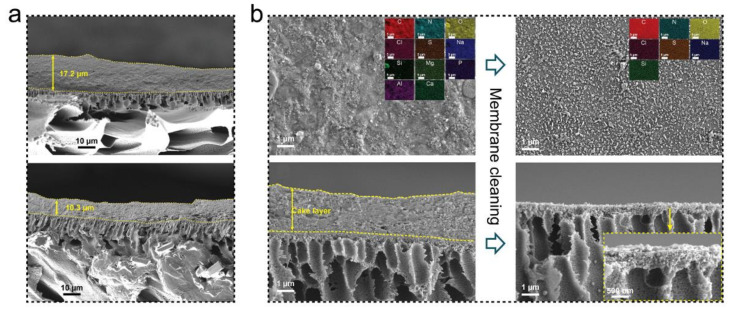
(**a**) SEM images of the cross-section of the FO (top) and GO-modified FO (bottom) membranes after 24 h filtration of sewage, and (**b**) surface (top) and cross-sectional (bottom) SEM of the GO-modified FO membrane before and after the last cleaning cycle, adapted from [33].

Another way to improve the antifouling property of FO membrane is membrane modification. Although membrane modification by inorganic or organic materials has been widely reported as an effective approach to improve FO membrane performance and properties, the application cases of modified FO membranes in wastewater treatment are few. Surface modification of FO membranes by polyethylenimine (PEI) effectively improved the ammonium rejection rate from 75.5% to 89.3% when treating return activated sludge (RAS), due to the enhanced surface hydrophilicity and positively charged PEI-modified FO membranes [52]. In another study, Zou et al. [53] reported a facile approach to fabricate a surface-modified TFC-FO membrane with zwitterion functionalised carbon nanotubes. The modified TFC-FO membrane showed reduced reverse solute flux due to more extended electrostatic repulsion in the presence of lower ionic strength solutions, improved fouling resistance, and stable water flux during the filtration of the secondary effluent from a local wastewater treatment plant. Graphene oxide (GO) is widely applied in membrane modification [16,54]. Wu et al. [33] reported a study about sewage concentration using GO-modified TFC-FO membranes. The result indicated that the GO-modified FO membrane showed 41% enhancement in water flux without reducing the selectivity property, due to the improved membrane hydrophilicity and reduced polyamide layer by adding GO nanosheets into the polyamide layer. When filtrating raw sewage, the GO-modified FO membrane exhibited a smaller water flux decline and a thinner foulant layer on the membrane surface (Figure 3a), which indicated the improved antifouling property. Moreover, during a continuing filtration experiment, the membrane fouling could further be controlled by a simple in-line water cleaning-in-place (CIP) process. Most of the pollutants (Mg, P, Al, Ca, and Si) could be washed off from the membrane surface (Figure 3b) and the GO-modified FO membrane achieved ~90% water flux recovery rate [33].

It should be mentioned that when designing the FO system for wastewater treatment, all factors elaborated above should be considered comprehensively, since these factors are interactive, and some potential trade-offs exist. For example, previous studies indicated that the energy consumption of the FO process could be reduced by system optimisation [3,7,28,37,55]. Iskander et al. [7] reported a study focusing on the energy perspective of the FO system for landfill leachate treatment. Their result indicated that the application of the osmotic backwashing process or chemical cleaning could effectively mitigate the membrane fouling and slow down the reduction of water flux. Compared to the benefit brought by membrane cleaning processes, its estimated energy consumption was negligible. Gulied et al. [28] found that the energy cost of the FO system can be reduced by increasing draw solution concentrations. In their work, they used a commercial fertiliser as the draw solute and their result indicated that the energy consumption of the FO system was reduced from 0.04 to 0.036 kWh/m^3^ by increasing the concentration of fertiliser from 100 to 200 g/L. However, a high concentration of draw solution also provides a negative impact on the FO system by causing the reverse salt flux. In order to improve the performance of FO membranes, the application of hydraulic pressure by increasing the flow velocity of feed solution was proposed [3,55]. Although a higher feed flow rate can reduce the ECP, enhance hydrodynamic conditions of fluid, and improve the mass transfer coefficient [28], it also aggravates membrane fouling by reinforcing the adhesion of foulants on membrane surface [37], and consequently increases the energy consumption of the FO system. Another factor that should be considered is the cost of FO modules. Due to the lower membrane packing density in a FO module compared to that in a RO module, the cost of a FO module was estimated at 55 USD/m^2^, which is higher than a RO module (24 USD/m^2^) [56,57,58,59].

## 3. The Integration of FO with Other Membrane Technologies

**Figure 4:**
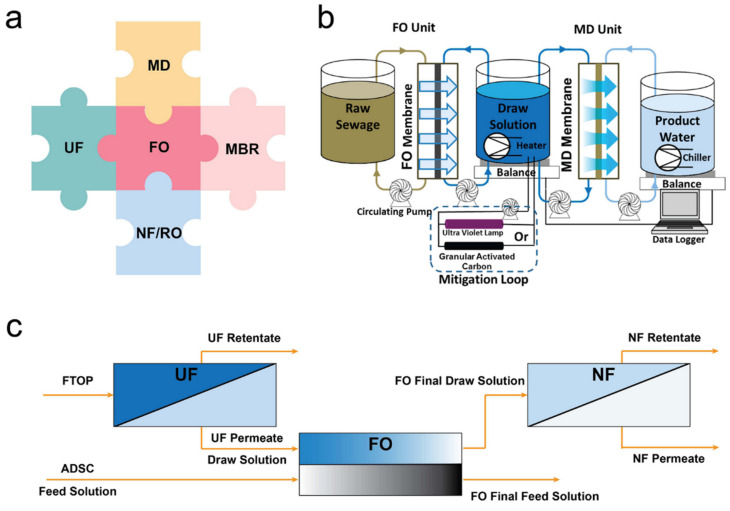
(**a**) Illustration of the integration of FO with other membrane-based technologies, (**b**) schematic diagram of the FO-MD hybrid system with the mitigation loop for the reduction of the concentration of feed contaminants in the draw solution, reprinted with permission from [61], and (**c**) schematic diagram of the UF-FO-NF integrated system, adapted from [62].

Current studies have revealed the feasibility of the FO filtration process in wastewater treatment. However, drawbacks such as the salt accumulation, requirement of draw solution recovery process, and further water mining from draw solution are still challenges that limit the wide application of stand-alone FO processes [60]. As a result, integrating the FO process with other membrane filtration processes is proposed (Figure 4a and Table 3). Some of the combinations are win-win strategies to apply the advantages of varied technologies and avoid their shortcomings.

### 3.1. Integration of FO with Membrane Distillation

Membrane distillation (MD) is an emerging separation process in water treatment. During the MD process, water molecules transfer from a high-temperature feed solution as vapor and dilute on the other side of the MD membrane. It has been reported that the MD process can completely reject non-volatile substances and has high salt selectivity [63]. The integration of FO with MD processes together can provide several advantages. Firstly, due to the fact that the FO process cannot produce clean water directly, the application of the MD process to recover clean water from diluted FO draw solution is a good strategy. Secondly, the draw solution for the FO process can be concentrated and recovered by the MD process [64]. Moreover, the FO process can be used to dilute the feed solution for the MD process, reducing the salt concentration and increasing the performance of the MD process.

Zhang et al. [64] reported a study about the application of a forward osmosis-membrane distillation (FO-MD) system for sustainable water recovery and acetic acid reuse from oily wastewater. Results indicated that the FO process showed a large water flux, a high oil removal ratio, and a moderate acetic acid permeation rate. After that, the MD process further rejected the NaCl and oil in diluted draw solution, while completed the regeneration of draw solution. The FO-MD system is also studied for treating wastewater from other sources. Industrial wastewater contains highly toxic heavy metals such as Hg, Cd, and Pb, and its discharge is strictly regulated [69,70]. Wu et al. [60] investigated the FO-MD system for the treatment of wastewater containing heavy metals. The result indicated that the FO system could effectively reject more than 97% of Hg, Cd, and Pb, and the MD system achieved around a 100% rejection rate of these heavy metals. Human urine consists of 95% water, 3.5% organics, and 1.5% inorganic salt [71], and is a potential water and nutrients source. Therefore, various technologies have been applied in water and nutrient recovery from urine, including electrodialysis, reverse osmosis, freeze/thaw concentration [72], microbial fuel cell, and ion exchange membrane [65,73,74]. Recently, Liu et al. [65] investigated the feasibility of a FO-MD hybrid system for treating urine. The FO membrane rejected most of the TOC, TN, and NH_4_^+^-N, but there were still some contaminants accumulated in the draw solution. Then, the MD process further rejected these contaminants, and the hybrid FO-MD system revealed nearly 100% rejection of TOC, TN, and NH_4_^+^-N. Recently, Liu et al. [65] investigated the feasibility of a FO-MD hybrid system for urine treatment. The FO membrane rejected most of the TOC, TN, and NH_4_^+^-N, but there were still some contaminants accumulated in the draw solution. Then, the MD process further rejected these contaminants, and the hybrid FO-MD system revealed nearly 100% rejection of TOC, TN, and NH_4_^+^-N (Table 4).

Although the hybrid FO-MD system combines the advantages of both FO and MD processes, there are still some drawbacks. In the FO-MD system, the MD membrane usually has higher selectivity than the previous FO membrane. As a result, contaminants that transfer from feed solution to FO draw solution but are rejected by the MD membranes will accumulate in the FO draw solution, which consequently decreases the quality of draw solution and leads to membrane fouling. For example, Xie et al. [61] observed an accumulation of trace organic contaminants (TrOCs) in the FO draw solution of the hybrid FO-MD system due to the significantly higher TrOCs selectivity of the MD membrane than that of the FO membrane. In order to mitigate the accumulation of TrOCs in the FO-MD hybrid system, Xie et al. applied granular-activated carbon (GAC) adsorption and ultraviolet (UV) oxidation to treat the draw solution (Figure 4b). The result indicated that both GAC adsorption and UV oxidation effectively mitigated the accumulation of organic matter and TrOCs in the FO-MD hybrid system.

### 3.2. Integration of FO with UF/NF/RO

Besides the combination with MD processes, the FO system also shows the feasibility to integrate with conventional pressure-driven membrane technologies, including UF, NF, and RO processes. The major aim of the FO process is to reduce the fouling tendency of UF, NF or RO membranes. Hafiz et al. [66] reported a study about a hybrid FO-RO system to produce water for irrigation. In this study, the efficiency of the FO process was evaluated by comparing two draw solutions, including NaCl solution (0.5 mol/L) and engineered fertilising solution (EFS, 0.5 mol/L NaCl and 0.01 mol/L diammonium phosphate ((NH_4_)_2_HPO_4_). Compared to the NaCl solution, the EFS solution has a higher osmotic pressure, which results in higher water flux in the FO process. However, compared to the energy consumption of the MBR-RO system (1.2–1.5 kW h/m^3^), the energy consumption for draw solution regeneration was higher in the FO-RO system (2.18–2.58 Kwh/m^3^). Toilet wastewater is the main source of municipal sewage pollutants. On the other hand, the human excreta contain a large amount of nutrients such as N, P, and K, which are crucial for the growth of plants, but are costly to eliminate in wastewater [75]. The commonly used end-of-pipe systems have high energy and maintenance costs [76]. Based on the hybrid FO-RO process, Xu et al. [77] designed a pilot-scale toilet wastewater treatment system to achieve a closed-loop cycle of water and nutrients from urine. In this system, the urine was diluted by flushing water, followed by being concentrated by the FO process 2.5 times. After that, the recovered flushing water was treated by the RO process for reuse of toilet flushing. Moreover, the concentrated urine can be used as a liquid fertiliser. Carbonell-Alcaina et al. [62] proposed a hybrid system containing UF, FO, and NF filtration processes to remove the organic matter in the effluent from a table olive fermentation process (FTOP) and dewater the anaerobically digested sludge concentrate (ADSC). In this system, FTOP was firstly filtrated by the UF membrane to remove organic matter. After that, the filtrated FTOP was applied as the draw solution for the FO process, and ADSC was applied as the feed solution (Figure 4c). After the FO treatment, the conductivity of FTOP was reduced, which was beneficial to the following NF process, and the ADSC was concentrated further. Their result indicated that the integrated process had high rejection (97%) of COD and phenolic compounds. Giagnorio et al. [78] reported a study to model the design of a full-scale FO-NF system for wastewater treatment. In this simulated case, the membrane area was approximately 7700–8500 m^2^ for FO membranes and 2900–3100 m^2^ for NF membranes. By using Na_2_SO_4_ or MgCl_2_ as the draw solutions and maintaining the feed solution at 76 m^3^/h, the FO-NF system can achieve 78–86% water recovery in the FO system and 61–65 m^3^/h water product from the further NF system. However, more research should be conducted to consider the energy efficiency and cost when using FO for wastewater treatment. It was reported that the cost of water production was as high as 132.0 USD/m^3^ when applying 2500 m^2^ FO membranes for leachate treatment [7].

### 3.3. Integration of FO with Membrane Bioreactor

A membrane bioreactor (MBR) such as an aerobic membrane bioreactor (AeMBR) and an anaerobic MBR (AnMBR) combines the biological process and membrane technologies and has several advantages, such as high solids removal rate, small energy consumption, and easiness for management [79]. However, the efficiency of MBR is limited by the low-strength property of wastewater. Therefore, the application of FO as the pre-concentration process can concentrate the wastewater and consequently improve the efficiency of MBR processes [80].

**Figure 5:**
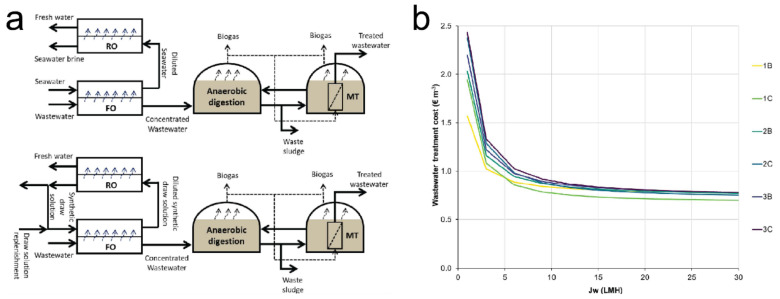
(**a**) Open-loop FO-RO-AnMBR system (top) and closed-loop FO-RO-AnMBR system (bottom), and (**b**) analysis of FO-RO-AnMBR cost in function to the FO membrane water flux, adapted with permission from [56].

Vinardell et al. [56] reported a study focusing on the economic analysis of a hybrid system integrating FO with AnMBR and RO processes. In this system, the FO was used to pre-concentrate the AnMBR influent, while the RO system was applied for draw solution regeneration and water production. In their study, the efficiency and economic cost of two draw solution management strategies including an open-loop and a closed-loop were compared (Figure 5a). The result indicated that the water flux and recovery rate of FO membranes were major factors to influence the economic cost of the system. Specifically, in the closed-loop, the economic cost increased from 0.81 to 1.27 EUR/m^3^ by increasing the FO recovery rate from 50% to 90%. The result also indicated that increasing the water flux of the FO membrane to 10 L/m^2^h could significantly reduce the wastewater treatment cost. However, it is interesting to find that when the water flux of the FO membrane was larger than 10 L/m^2^h, the wastewater treatment cost reduced slightly (Figure 5b). Therefore, although the high water flux is one of the major scientific goals in FO technology, it still needs to consider whether the impact of high water flux is overestimated when applying the FO membrane in industrial applications.

## 4. Integration of FO with Other Wastewater Treatment Technologies

**Figure 6:**
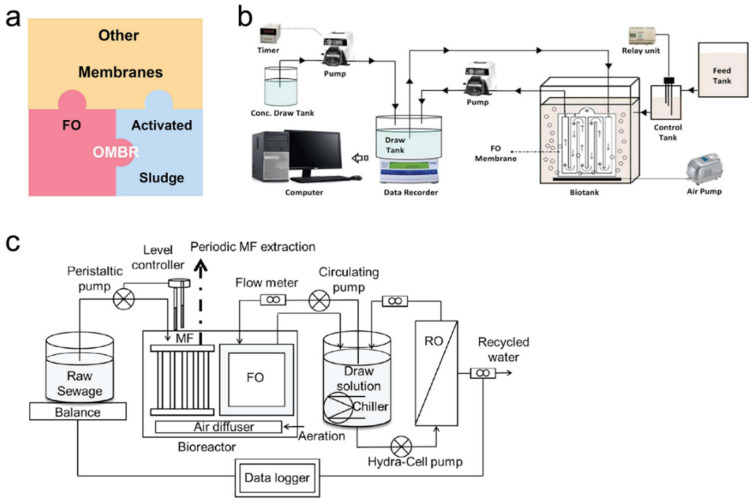
(**a**) Illustration of the integration of OMBR with other membrane technologies, (**b**) the submerged OMBR system, reprinted with permission from [81], and (**c**) the MF-OMBR–RO hybrid system, reprinted with permission from [91].

**Figure 7:**
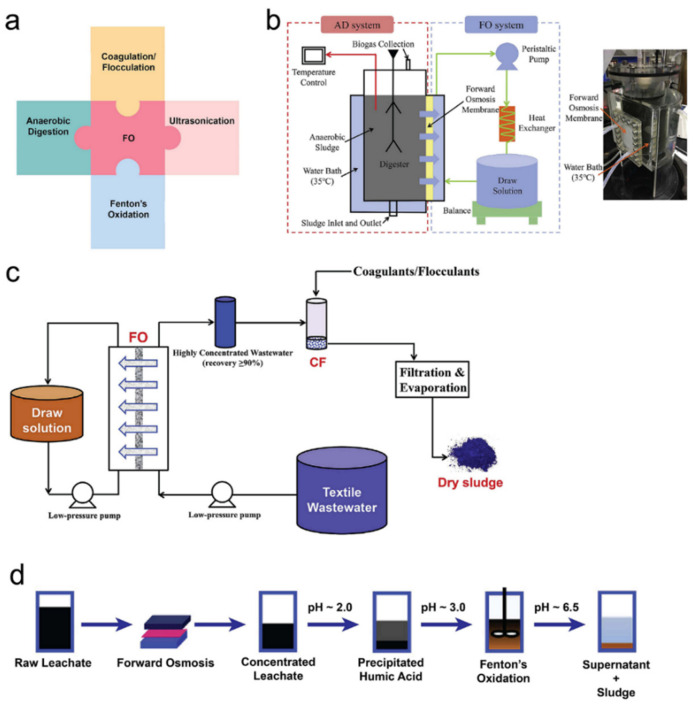
(**a**) Illustration of the integration of FO with other water treatment technologies, (**b**) the hybrid FO-AD system, reprinted with permission from [105], (**c**) the hybrid FO-CF system, reprinted with permission from [113], and (**d**) the integration of FO with Fenton’s oxidation, adapted from [114].

Besides integrating with other membrane-based technologies, the FO process can also be applied as pre-treatment or post-treatment for other wastewater treatment processes. In this section, the integration of the FO process with activated sludge (Figure 6a), AD, CF, Fenton’s oxidation, and ultrasonication (Figure 7a) will be discussed. Moreover, an advanced perspective about reverse salt flux is involved.

### 4.1. Integration of FO with Biological Process

Apart from using FO as the pre-treatment process for MBR, another integration form between the FO and the biological process (i.e., activated sludge) is the osmotic membrane bioreactor (OMBR) (Figure 6a). In an OMBR system, the FO membrane is submerged inside the MBR and replaces conventional MF and UF membranes, as shown in Figure 6b [67,81]. The driving force in OMBR is osmotic pressure rather than external pressure or suction in MBR. Due to the application of the FO membrane, the OMBR system shows advantages including low fouling tendency and high-quality product water [82,83]. Moreover, the high selectivity property of the FO membrane can effectively concentrate salts, nutrients, and organic matter in the bioreactor, which can improve the efficiency of aerobic or anaerobic treatments [84,85,86,87,88]. Several studies have revealed the feasibility of OMBR systems for treating different wastewater. Qiu et al. [84] reported a study focusing on phosphorus recovery from municipal wastewater by the OMBR system. During the operation, the FO membrane effectively increased the concentrations of PO_4_^3−^, Ca^2+^, and Mg^2+^, while the organic matter and NH_4_^+^ were removed through the biological activities in the bioreactor. By controlling the pH at 9.0, the system recovered 95% of PO_4_^3−^ through the precipitation between PO_4_^3−^ and Ca^2+^, Mg^2+^, as well as NH_4_^+^. Additionally, during the 84-day continuing operation, the phosphorus recovery efficiency was around 50%. In another study, Yao et al. [67] investigated the removal and degradation mechanism of carbamazepine (CBZ) from wastewater through the application of OMBR. The result indicated that the OMBR system showed a high removal efficiency of COD (94.8%), NH_4_^+^-N (93.6%), and CBZ (88.2%). Chen et al. [89] reported that the AnOMBR process was more effective than the traditional AnMBR process and demonstrated good removal efficiency of organic carbon (96%) and TP (~100%). Gao et al. [90] also reported a study about the application of AnOMBR for water recovery from real municipal sewage, and results indicated that the AnOMBR system had great removal efficiency of COD (96%), NH_4_^+^-N (88%), TN (89%), and TP (~100%).

There are still several shortcomings of OMBR systems. Firstly, due to the reverse salt flux from NaCl draw solution and the concertation effect of wastewater by the FO process, salt is accumulated in the reactor [85,86,92]. The accumulated salt can reduce the osmotic pressure differences across the membrane as well as increase the density and viscosity of wastewater, consequently resulting in lower water flux of the FO process [93,94]. It also aggravates membrane fouling and damages the condition of activated sludge for microorganisms [80,90,94]. In order to mitigate the salt accumulation in OMBR systems, several strategies were proposed in current studies, including the selection of suitable draw solution with lower reserve salt flux [29], the fabrication of high-performance FO membranes [95], and the combination of MF/UF technologies with OMBR [96].

Luján-Facundo et al. [29] investigated the feasibility of the application of real wastewater solution from an absorption column consisting mainly of SO_4_^2−^ and NH_4_^+^-N as the draw solution for the OMBR system. Before application, the draw solution was adjusted at 4.0 pH to avoid the chemical damage of FO membranes. Their result indicated that the OMBR system showed low membrane fouling tendency and high water flux with this draw solution. In another study by Luján-Facundo et al. [97], they used the same wastewater as the draw solution to treat tannery wastewater which contained high salinity and organic matter concentration in an OMBR system. Although the reverse salt flux was mitigated by using this new draw solution, the salt accumulation was also observed due to the dewatering process of the naturally high-salinity wastewater. As a result, the efficiency of OMBR and bacterial activity was still significantly affected.

Another effective strategy proposed to mitigate the salt accumulation in the OMBR process is the integration of OMBR with other membrane filtration technology, such as MF, UF, and RO processes. By submerging MF or UF membranes into the bioreactor of the OMBR system, the salt in the bioreactor can be removed by transferring through the MF and UF membranes. As a result, the salinity accumulation was mitigated [98]. Qiu et al. [68] exhibited the application of a MF/OMBR system in their study about phosphorus recovery from municipal wastewater. After the dewatering by the FO process, the wastewater was enriched in the bioreactor. Using the MF membrane, the phosphorous was recovered by extracting the supernatant in the bioreactor. Their results indicated that the hybrid system demonstrated high removal efficiency of TOC (90%) and NH_4_^+^-N (99%), as well as high phosphorus recovery (>90%). The long-term stability of the MF-OMBR system was evaluated by Luo et al. [99] in a 60-day continuing study, which further indicated that the MF membrane can remove the salts from the bioreactor of the OMBR system and prevent salinity accumulation.

Despite the advantages of the MF/UF-OMBR system, there are some challenges. For example, in a study about TOC and NH_4_^+^-N removals by a MF-OMBR system, Wang et al. [100] observed the membrane fouling, especially, the reversible fouling in the MF-OMBR was more serious than that in the OMBR system, which led to increased filtration resistance, aggravated ECP, and declined water flux. In addition, the fouling mitigation for the MF/UF-OMBR system may be complex due to the different fouling cleaning strategies between MF/UF (hydraulic backflushing) and FO membranes (osmotic backflushing) [98]. Another drawback of the MF/UF-OMBR system is a lack of technology to regenerate draw solutions. Therefore, Luo et al. [91] investigated a hybrid system integrating MF-OMBR and RO processes (Figure 6c). In this hybrid system, the MF membrane was applied to remove phosphorus from the enriched wastewater and to mitigate the salinity build-up in the bioreactor, while the RO process was for draw solute recovery and clean water production. The result indicated that the hybrid system can produce high-quality water. However, the accumulated organic matter and ammonia in the FO draw solution is a challenge requiring further investigation.

Similar to the stand-alone FO process, the efficiency of FO-integrated systems is impacted by factors such as the draw solution type, draw solution concentration, and cross-flow velocity. However, it is more complex when considering the optimal operating condition for FO-integrated systems since more factors in different treatment processes should be considered. Two of the major energy consumers of the FO process are draw solution reconcentration and recirculation pumps [101]. Cath et al. [102] estimated the specific energy consumption (SEC) of a FO-RO system. Their result indicated that 19 kWh/m^3^ was required for solute reconcentration by the RO system, which accounted for 76% of the total SEC of the FO-RO system. Therefore, improving the solute reconcentration efficiency is critical to reduce energy consumption. McGinnis et al. [103] reported a study by using NH_3_-CO_2_ solution as the draw solution for the FO process. The diluted NH_3_-CO_2_ solution can be reconcentrated by waste heat, which reduced the energy consumption of the system to 0.84 kWh/m^3^. Recirculation pumps also consumed a large proportion of the SEC, which was estimated at 25–30% [101]. He et al. [7,104] found that the energy consumption for recirculation pumps could be reduced by decreasing the recirculation flow rate. However, the reduced water flux of the FO membrane and increased fouling caused by a lower recirculation flow rate should be considered when determining an optimal recirculation flow rate. Park et al. [98] reported a model to find optimal design parameters for the OMBR-RO system. The result indicated that increasing flow rates and concentrations of draw solution could improve the water flux of FO membranes, and could consequently reduce the cost of purchasing FO membrane, but it would also increase energy consumption of the FO system. Vinardell et al. [56] evaluated the feasibility of retrofitting the RO plant (final water production of 45,000 m^3^/day) to a FO-RO-MBR plant. The result indicated that the FO-RO-MBR system was economically competitive if the recovery rate of the FO system was maintained at 50%. When the recovery rate increased to 80% or higher, the cost of the hybrid system was larger than that of the standalone RO plant. Therefore, taking into account the factors of both FO membranes and FO systems is critical for a highly efficient FO process.

### 4.2. Integrations of FO with Other Water Treatment Processes

Anaerobic digestion (AD) is an effective method to treat the waste sludge from wastewater treatment plants. However, due to the long retention time and low-solid concentration in the reactor, AD has a relatively high construction cost for building a large reactor [105]. It has been revealed that using concentrated sludge as the feeding substrate and applying high-solid AD can improve the efficiency and reduce the cost [89,106,107]. Gao et al. [108] reported a system integrating FO with AD processes. Results indicated that the FO process can be an effective pre-treatment process to concentrate sewage which can then be used as the influent for the AD process. Zhao et al. [105] reported a study about integrating OMBR and AD processes. Distinguished from the research by Gao et al. [108], the feed solution dewatering happened at the same time as the AD process in Zhao’s system (Figure 7b). The hybrid AD-OMBR system showed better performance than the conventional AD system, with high solid content, organic degradation, and methane content in biogas. Textile wastewater is one of the most polluting wastewaters [109]. The CF process is an effective approach for textile wastewater decolourisation [110,111]. Similar to the AD process, the efficiency of the CF process can be improved by using concentrated dye wastewater [112]. Han et al. [113] reported a FO-CF hybrid system to treat textile wastewater (Figure 7c). In this system, the FO membrane showed a 99.9% rejection rate of dye, and effectively reduced the volume of wastewater. Due to the successful enrichment of textile, the further CF process exhibited a high dye removal rate (>95%) by using 500−1000 ppm of coagulants and flocculants.

Fenton’s oxidation is a process that can be used to remove COD and TOC from landfill leachate [115,116]. However, Fenton’s oxidation process requires a strict pH condition between 2 and 4 [115,117], resulting in a large amount of reagent dosage such as H_2_SO_4_. In order to reduce the reagent dosage and enhance the efficiency of Fenton’s oxidation, Iskander et al. [114] designed a system integrating FO with humic acid (HA) recovery and Fenton’s oxidation technologies. In this integrated system (Figure 7d), the FO process was applied to reduce the volume and alkalinity of leachate by dewatering and the concentration gradient-driven movement of the alkalinity-causing species, respectively. As a result, the required amount of H_2_SO_4_ for maintaining pH was reduced. After the FO process, the HA recovery process was applied to remove the humid substances from the wastewater, which provided positive effects to reduce the reagent (Fe (II) and H_2_O_2_) for further Fenton’s oxidation. Moreover, the recovered HA can be used to remove aqueous phosphorus, nitrogen, heavy materials by precipitation, and can be a type of fertiliser [114,118,119]. Compared to the single Fenton’s oxidation process, the integrated system reduced the required amount of H_2_SO_4_ by 25.2%, NaOH by 34.6%, and H_2_O_2_ by 35%.

In another study, Nguyen et al. [120] proposed a hybrid system that integrated ultrasonication and OMBR technologies to further improve the efficiency for sludge disintegration and dewater of OMBR systems. The result showed that the application of ultrasonication could improve the sludge concentration performance of OMBR. Specifically, to increase the sludge concentration from 3000 to 20,400 mg/L, the conventional OMBR needed 26 h, while the ultrasonication OMBR system only required 22 h. Furthermore, the ultrasonication OMBR system could achieve NH_4_^+^-N removal efficiency of 96%, PO_4_^−3^ of 98%, and dissolved organic carbon (DOC) of 99%. The major reason for this improvement was due to that the ultrasonic process affected the sludge solubilisation and reduced floc size, which facilitated the release of organic substances and bounded water into the liquid phase. Moreover, the application of ultrasound mitigated the membrane fouling by hindering the adhesion of foulants on the membrane surface.

## 5. Emerging Application of FO for Resource Recovery

**Figure 8:**
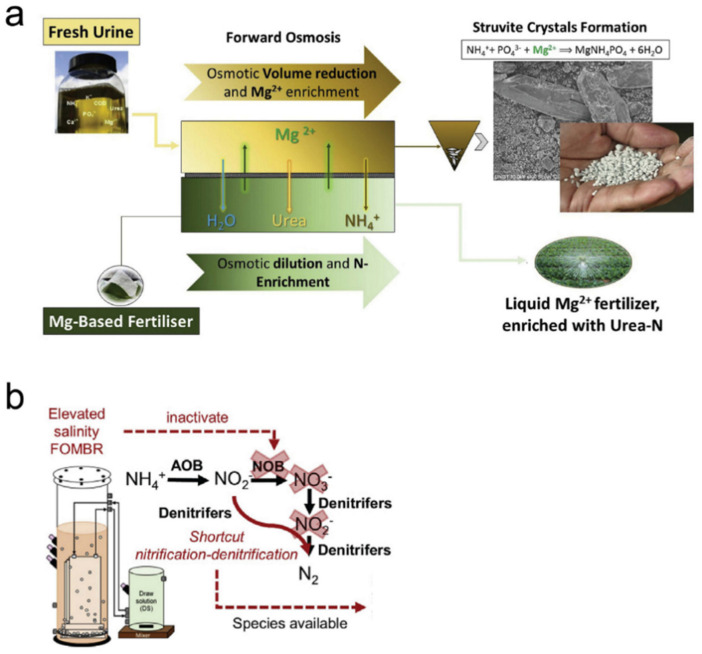
(**a**) Schematic diagram of the FO process for phosphorous and nitrogen recovery from urine, reprinted with permission from [121], and (**b**) zchematic diagram of a nitrification-denitrification shortcut built in FOMBR system, reprinted with permission from [88].

Reverse salt diffusion from draw solution to feed solution has generally been considered as a drawback during the FO process, because it can reduce the osmotic pressure of draw solution and pollute the biological condition in the bioreactor. However, by integrating FO with other technologies, reverse salt diffusion can be applied in a positive way. Volpin et al. [121] proposed a new strategy for phosphorous and nitrogen from urine by using the FO process (Figure 8a). They used Mg^2+^-based fertiliser as the draw solution to recover water and urea/NH_3_ from urine. Due to the reverse Mg^2+^ diffusion from draw solution to the urine, the phosphorous recovery can be achieved through struvite crystals’ formation. In addition, water and NH_4_^+^ from urine can reduce the volume of urine and enrich the fertiliser at the same time. Another example of the utilisation of reverse salt diffusion is the creation of a nitrification-denitrification shortcut (Figure 8b) [88]. In a general aerobic-anoxic system, NH_4_^+^ was firstly oxidised by ammonia-oxidising bacteria (AOB) to form NO_2_^−^, and then was transferred to NO_3_^−^ by nitrite-oxidising bacteria (NOB). After that, the NO_3_^−^ was denitrified to NO_2_^−^ and further reduced to N_2_ [122]. However, when increasing the salinity, the activity of NOB can be impacted, while the denitrifies were not influenced [94]. Therefore, increasing the salinity can provide a shortcut of the nitrification-denitrification process, resulting in a direct formation of N_2_ from NO_2_^−^, which can reduce biomass production. Hamid et al. [88] reported a 243-day OMBR experiment to treat municipal wastewater. Due to the reverse NaCl diffusion, the salinity in the feed solution was elevated gradually. As a result, a shortcut nitrification-denitrification process was achieved after 72 days. When changing the draw solution from 35 to 70 g/L, the OMBR system kept running. At the end of the 243-day operation, the FO membrane still had a 67% water recovery rate. The AOB (*Nitrosomonas sp.*) was the only nitrifier species, which further confirmed the building of a nitrification-denitrification shortcut in the system.

## 6. Conclusion and Future Prospects

Current studies indicate that both the concentration of feed solutions and the dilution of draw solutions in the FO process can be utilised for wastewater treatment, which expand the application areas of the FO process. Due to advantages such as high selectivity and a low fouling tendency, the FO process has been applied to concentrate various types of wastewater such as sewage, oil sands process-affected water, and radioactive wastewater for the purposes of recovering water, concentrating nitrogen, and extracting dye. On the other hand, by using the dilution effect of the FO process, wastewater/sewage can be used to dilute fertilisers. The FO process also shows good performance in combination with other membrane-based technologies and biological/chemical technologies, such as MBR, AD, and CF to increase the treatment effect. Additionally, the reverse salt diffusion can be applied in a positive way to trigger the phosphorus precipitation and to create a nitrification-denitrification shortcut. However, there are still many challenges in the current FO technology in the areas including membrane materials, draw solution selection, and system optimisation.

Although membrane modification by nanomaterials has been reported as an effective way to improve the performance and antifouling properties of FO membranes, most of the previous works were done in lab-scale and rarely in real wastewater treatment. Therefore, the stability and antifouling ability of the nanomaterials-modified FO membranes need more investigation on a large scale or during a long-term operation. There are a few cases associated with surface modification of FO membranes for wastewater treatment, which shows the potential to increase the antifouling property of FO membranes. However, surface modification cannot address the issues caused by ICP. The ICP not only aggravates the membrane fouling but also reduces the efficiency of FO systems, especially when treated with highly polluted feed solution. Current TFC-FO membranes usually apply UF as the substrate, which has a relatively low porosity. Although increasing the porosity of substrates of the TFC-FO membrane can mitigate ICP, the impacts of porous substrates on the formation of selectivity layer of TFC-FO membranes, mechanical, and chemical strengths, especially in highly acidic conditions, need more investigation.

**Figure 9:**
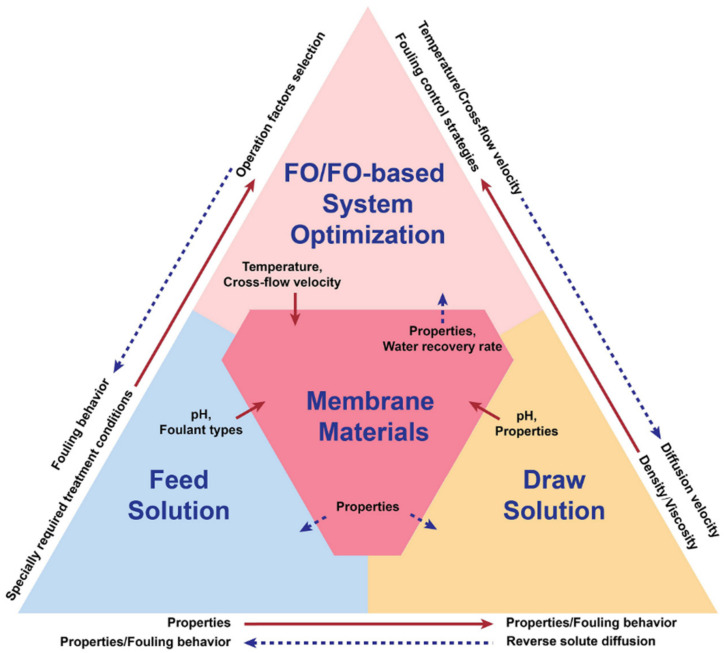
Schematic diagram of the relationships among membrane materials, draw solution, feed solution, and system optimisation in single FO or FO-integrated wastewater treatment processes.

A suitable draw solute is very important for both single FO and FO-integrated systems. Some new draw solutes with good osmotic pressure and small reverse solute diffusion have been implemented in cases highlighted in this review. However, a more comprehensive evaluation of these draw solutions or other new draw solutions should be conducted in terms of price, recovery cost, impacts on membrane fouling, and impacts on the chemical stability of the feed solution. Another important consideration is the properties of the feed solution. The different discharge standards of feed solutions should be considered in the design of FO-based wastewater treatment systems. Moreover, system factors such as the temperature, cross-flow velocity, and cleaning process are interactive and work together to determine the efficiency and energy cost of FO systems. Therefore, it is crucial to consider the synergistic effect and trade-offs among these factors. When integrating the FO process with other technologies, there are more interactions among membrane materials, feed solution, draw solution, and system-operating conditions, as summarised in Figure 9. For example, the chemical properties of draw solutions such as the pH may affect the choice of membrane materials, and the density and viscosity of draw solution can be influenced by the system temperature. The operation conditions restrict the membrane properties such as mechanical stability, while the properties of feed solutions may also constrain the process of membrane fabrication, such as requiring the membranes to have certain special properties such as chlorine resistance and organic solvent resistance. Therefore, when designing a FO-integrated system, all factors should be considered to achieve an optimal operation condition.

## Figures and Tables

**Table 1:** Application of the FO process as a single treatment process for sewage concentration and wastewater treatment.

Membrane	Feed Solution	Draw Solution	Membrane Area	Temperature (°C)	Flow Rate/Velocity	Operation Time	Water Flux (LMH)	Filtration Performance	Ref.
CTA-FO	Municipal wastewater	0.5–4.5 mol/L NaCl	124 cm^2^	20	375 L/h	6–7 h	4.3–12.5	-	[3]
CTA-FO	Municipal wastewater	0.5 mol/L NaCl	0.3 m^2^	18–22	20 cm/s	51 days	~5.0	Rejection: COD (99.8%), TP (99.7%), NH_4_^+^ (48.1%), and TN (67.8%)	[4]
CTA-FO	THP-AD sludge filtrate ^1^	2 mol/L NaCl	195 cm^2^	25	90 L/h	20 h	1.0–6.0	Rejection: Total organic carbon (TOC, 93.0%), TN (92.8%), NH_4_^+^-N (92.5%), TP (96.5%), Fe (99.0%), Mn (99.0%), Ca (95.7%), and Mg (97.1%)	[35]
Aquaporin embedded TFC-FO	Molasses distillery wastewater	3 mol/L MgCl_2_·6H_2_O	43 cm^2^	-	108 L/h	24 h	2.7	Rejection: COD (85.2%), melanoidins (97.3%), and antioxidant activity (94.2%)Water recovery: 65%	[36]
CTA-FO	Simulated radioactive wastewater	2 mol/L NaCl	40.5 cm^2^	25 ± 2	2–11 cm/s	3 h	15.3 (ALFS) and 19.3 (ALDS)	Ion flux in ALFS mode: Co (1.54 mg/m^2^h), Sr (10.22 mg/m^2^h), and Cs (15.63 mg/m^2^h)	[23]
CTA-FO	OSPW ^2^	4 mol/L NH_4_HCO_3_	64 cm^2^	21± 1	1.26 L/h	28 h	68.1 (Max)	Rejection: F^−^, NO_2_^−^,Br^−^, Al, Ca, Fe, Sr, Mo and Ba (>70%) Water recovery: 85%	[37]
CTA-FO	ADSC ^3^	Industrial effluent ^4^	42 cm^2^	-	30 L/h	70 h	2.5–4.0	Concentration factor: NH_4_^+^-N (1.42)	[38]
TFC-FO	Municipal wastewater	Synthetic seawater concentrate	20.02 cm^2^	35	16.8 L/h	24 h	~15.5–18.5	Concentration factor: COD (2.5), NH_4_^+^-N (1.5), TN (1.75) and TP (3.4)	[34]

^1^ Anaerobically digested sludge filtrate after thermal hydrolysis pretreatment. ^2^ Oil sands process-affected water. ^3^ Anaerobically digested sludge concentrate. ^4^ Residual stream from an absorption process for ammonia elimination.

**Table 2:** The physicochemical properties of raw municipal wastewater and concentrated wastewater after the FO process [34].

Wastewater	COD (mg/L)	TP (mg/L)	TN (mg/L)	NH_4_^+^-N (mg/L)
Raw municipal wastewater	165–229	1.69–2.74	30.5–44.8	24.6–37.5
Concentrated wastewater after FO process	438–563	5.92–9.37	55.4–83.5	43.2–63.0

**Table 3:** The integration of FO with other water treatment technologies.

Integrated System	Feed Solution	Draw Solution	Membrane Area	Temperature (°C)	Cross-Flow Rate/Velocity	Operation Time of FO	Water Flux (LMH)	Filtration Performance	Ref.
FO-MD	Simulated wastewater containing HgCl_2_, Pb(NO_3_)_2_, and CdCl_2_	1 mol/L NaCl	42 cm^2^ (FO) and 100 cm^2^ (MD)	~20 (FO) and ~55 (MD)	6 L/h (FO) and 90 L/h (MD)	5 h	~6.1	Hg rejection: >97 % (FO) and ~100% (FO-MD)	[60]
FO-MD	Human urine	2 mol/L NaCl	29.5 cm^2^ (FO) and 29.5 cm^2^ (MD)	25 (FO) and 50 (MD)	12 L/h	8 h	~5.5	Urine concentration factor: 1.116 Water production rate: 10.385%	[65]
FO-RO	Treated sewage effluent (TSE) after a membrane bioreactor (MBR) unit	Engineered fertilising solutions (EFS) ^1^	42 cm^2^ (FO) and 42 cm^2^ (RO)	-	120 L/h	3 h	~13	Rejection: TP (99%) and NH_4_^+^ (95%)	[66]
UF-FO-NF	ADSC ^2^	Effluent from a table olive fermentation process (FTOP)	0.025 m^2^ (UF), 0.5 m^2^ (FO) and 0.0047 m^2^ (NF)	25	2.2 m/s (UF and NF) 42 and 250 L/h for the FO draw and feed solution respectively	10 h	4.0–5.5	Rejection: COD (88.7%), TN (58.1%), TP (100%) and colour (99.9%)	[62]
OMBR	Carbamazepine solution (50 μg/L, 100 μg/L, and 200 μg/L)	1 mol/L NaCl	50 cm^2^	26 ± 0.5	10 cm/s for draw solution	80 days	1.9–11.9	Rejection: COD (94.77–97.45%), NH_4_^+^-N (93.56–99.28%), and CBZ (88.20–94.45%)	[67]
MF-OMBR	Activated sludge	Seawater brine from desalination plant	0.072 m^2^	~20	-	98 days	7–9	Rejection: TOC (90.0%), NH_4_^+^-N (99.0%), and TP (>90.0%)	[68]

^1^ The engineered fertilising solutions (EFS) contain 0.5 mol/L NaCl and 0.01 mol/L diammonium phosphate. ^2^ Anaerobically digested sludge concentrate.

**Table 4:** The physicochemical properties of raw human urine and product water by the FO-MD process [65].

Wastewater	TOC (mg/L)	TN (mg/L)	NH_4_^+^-N (mg/L)	TP (mg/L)
Raw human urine	5298 ± 792	7523 ± 1097	1125 ± 147	448 ± 56
Product water by FO-MD	2.25 ± 0.04	0.2125 ± 0.0089	0.061 ± 0.006	-

## Data Availability

Not applicable.
